# Oxidative Stress Induces Endothelial Cell Senescence via Downregulation of Sirt6

**DOI:** 10.1155/2014/902842

**Published:** 2014-08-05

**Authors:** Rong Liu, Hua Liu, Yonju Ha, Ronald G. Tilton, Wenbo Zhang

**Affiliations:** ^1^Department of Ophthalmology, Tongji Hospital, Tongji Medical College, Huazhong University of Science and Technology, Wuhan 430030, China; ^2^Department of Ophthalmology and Visual Sciences, The University of Texas Medical Branch, 301 University Boulevard, Galveston, TX 77555, USA; ^3^Center for Biomedical Engineering, The University of Texas Medical Branch, Galveston, TX, USA; ^4^Internal Medicine, Division of Endocrinology and Stark Diabetes Center, The University of Texas Medical Branch, Galveston, TX, USA; ^5^Neuroscience and Cell Biology, The University of Texas Medical Branch, Galveston, TX, USA

## Abstract

Accumulating evidence has shown that diabetes accelerates aging and endothelial cell senescence is involved in the pathogenesis of diabetic vascular complications, including diabetic retinopathy. Oxidative stress is recognized as a key factor in the induction of endothelial senescence and diabetic retinopathy. However, specific mechanisms involved in oxidative stress-induced endothelial senescence have not been elucidated. We hypothesized that Sirt6, which is a nuclear, chromatin-bound protein critically involved in many pathophysiologic processes such as aging and inflammation, may have a role in oxidative stress-induced vascular cell senescence. Measurement of Sirt6 expression in human endothelial cells revealed that H_2_O_2_ treatment significantly reduced Sirt6 protein. The loss of Sirt6 was associated with an induction of a senescence phenotype in endothelial cells, including decreased cell growth, proliferation and angiogenic ability, and increased expression of senescence-associated *β*-galactosidase activity. Additionally, H_2_O_2_ treatment reduced eNOS expression, enhanced p21 expression, and dephosphorylated (activated) retinoblastoma (Rb) protein. All of these alternations were attenuated by overexpression of Sirt6, while partial knockdown of Sirt6 expression by siRNA mimicked the effect of H_2_O_2_. In conclusion, these results suggest that Sirt6 is a critical regulator of endothelial senescence and oxidative stress-induced downregulation of Sirt6 is likely involved in the pathogenesis of diabetic retinopathy.

## 1. Introduction

Increasing evidence indicates that diabetes accelerates the process of aging, especially in patients who are at high risk of developing complications [1]. Diabetic retinopathy (DR) is one of the most common complications of diabetes. It is a leading cause of blindness in people of working age in industrialized countries [2]. The mechanisms by which elevated blood glucose causes tissue injury and disease progression in the retina are not yet clear. However, studies in animal models and patients suggest that diabetic retinopathy is associated with vascular dysfunction, similar to changes seen during aging [3, 4]. Increased cellular senescence was also observed in retinal blood vessels from diabetic animals [4].

Aging is a normal process of any living mammal. The proliferative lifespan of primary mammalian cells is limited. After a given number of replication cycles, they enter permanent cell-cycle arrest, referred to as replicative senescence and linked to a reduction in telomerase activity and telomere shortening [5, 6]. In contrast to replicative senescence, stress-induced premature senescence is elicited by stressful stimuli and does not require telomere shortening and extensive cell division [5, 7]. Although caused by different mechanisms, replicative senescence and stress-induced premature senescence share many similarities, including a specific set of alternations in cell function, morphology, gene expression, and positive staining for senescence-associated *β*-galactosidase activity (SA *β*-gal) [7].

Premature senescence of endothelial cells is recognized to play a key role in the pathogenesis of diabetic vascular complications including diabetic retinopathy [4, 8, 9]. When endothelial cells acquire a senescent phenotype, their homeostatic functions become impaired as indicated by a decrease in production of nitric oxide and an increase in expression of adhesion molecules such as ICAM-1 [10]. These alternations lead to endothelial dysfunction, vascular inflammation, and impaired angiogenesis and vascular repair [9]. Endothelial senescence can be induced by a variety of factors; among them, oxidative stress has a major role [11].

Oxidative stress occurs when the production of reactive oxygen species (ROS) overwhelms endogenous antioxidant systems and/or when the endogenous antioxidant systems are impaired. ROS are a group of oxygen-based molecules characterized by their high chemical reactivity [12, 13]. ROS include free radicals such as superoxide (O_2_^−^) and hydroxyl radicals (OH^∙^), and nonradical species such as hydrogen peroxide (H_2_O_2_) [12, 13]. Excessive production of ROS critically contributes to development of many signs of DR ranging from vascular dysfunction and vascular leakage to pathological angiogenesis [2, 14–19]. Although oxidative stress is one of the major factors causing the onset of senescence [20], the specific mechanisms underlying ROS-induced endothelial senescence are not completely clear.

Mammalian sirtuins (Sirts) are the homologue of the yeast silent information regulator (Sir) 2, a nicotinamide adenine dinucleotide- (NAD-) dependent histone deacetylase regulating life span of yeast [21]. Among the seven mammalian Sirts, Sirt6 most closely resembles the yeast Sir2 regarding its intracellular location and function and the animal phenotype caused by loss of Sirt6 [21, 22]. It is a nuclear, chromatin-bound protein that functions as a NAD-dependent histone H3 lysine 9 (H3 K9) deacetylase, repressing the activities of several transcription factors involved in aging and inflammation, including NF-*κ*B, c-JUN, and hypoxia-inducible factor- (HIF-) 1*α* [23–26]. Mice deficient in Sirt6 exhibit severe metabolic defects and premature aging phenotype and 60% of them die at about 4 weeks of age [21]. In this study, we determined the role of Sirt6 in ROS-induced endothelial cell senescence.

## 2. Materials and Methods

### 2.1. Cell Culture and Transfection

Human umbilical vein endothelial cells (HUVECs) were purchased from Lonza (Lonza Walkersville Inc., Walkersville, MD) and grown in EGM Endothelial Cell Medium (Lonza). Cells between passages 4 and 7 were used for all experiments. To overexpress Sirt6, cells were infected with adenovirus carrying empty vector (control, Ad-Con) or Sirt6 (Ad-Sirt6) as described previously [27]. These adenoviruses were purchased from Vector BioLabs (Philadelphia, PA). One day after infection, HUVECs were treated with 75 *μ*M H_2_O_2_ (EMD Millipore, Billerica, MA) for 2–4 days, with medium being changed daily. To moderately knock down Sirt6, cells were transfected using HiPerFect (Qiagen, Valencia, CA) following the manufacturer's instructions. Human Sirt6 siGENOME SMARTpool consisting of a mixture of four sequences and a control nontargeting siRNA were purchased from Thermo Fisher Scientific Inc. (Waltham, MA) and used at a concentration of 34 nM. The transfection complexes were removed 24 hours later. Four and eight days after initiating transfection, cells were subjected to two additional transfections.

### 2.2. Immunocytochemistry

HUVECs were seeded on fibronectin-coated slides and infected with adenovirus carrying Sirt6 (Ad-Sirt6). At 24 hours after infection, cells were washed with Hank's Balanced Salt Solution (HBSS) and fixed in 2% paraformaldehyde-Phosphate Buffered Saline (PBS) for 15 minutes at room temperature. After washing with PBS, cells were permeabilized with 0.1% Triton-PBS for 5 minutes, blocked with PBS containing 2% bovine serum albumin (BSA) for 30 minutes, followed by incubation with primary antibody to Sirt6 (1 : 100, Cell Signaling Technology, Danvers, MA) in PBS containing 1% BSA for 2 hours at 4°C. After washing three times with PBS containing 0.1% BSA, cells were incubated with secondary antibody Alexa Fluor 488 donkey anti-rabbit (1 : 250, Invitrogen, Carlsbad, CA) for 1 hour at 4°C and washed and mounted with Vectashield mounting medium containing DAPI (Vector Laboratories, Burlingame, CA). Slides were imaged using an Olympus 1X71 fluorescence microscope (Olympus, Center Valley, PA) at a magnification of 100x.

### 2.3. Senescence-Associated *β*-Galactosidase (SA-*β*-gal) Staining

Senescence-associated *β*-galactosidase (SA-*β*-gal) staining was performed using a Senescence Detection kit (BioVision, Milpitas, CA) according to the manufacturer's instructions. Briefly, cells were washed with HBSS, fixed using a fixative solution for 10–15 minutes, again washed with PBS, and then incubated with Staining Solution Mix overnight at 37°C. Cells were imaged under a bright-field microscope at a magnification of 100x. The number of positive cells with blue color was counted and normalized to the number of total cells in the same field.

### 2.4. Western Blot

Western blots were performed as described [27]. Briefly, cells were lysed in SDS sample buffer without dithiothreitol and bromophenol blue and then cleared of debris by centrifugation. Protein concentration in the lysates was determined by a bicinchoninic acid (BCA) assay (Pierce Biotechnology, Rockford, IL). Dithiothreitol was added to a final concentration of 125 mM and samples were boiled for 10 minutes. Equal amounts of protein were subjected to 10% SDS-PAGE, transferred onto nitrocellulose membranes, and the membranes were incubated overnight with one of the following primary antibodies: anti-Sirt6 (1 : 1000), anti-eNOS (1 : 1000, BD Biosciences, San Jose, CA), anti-tubulin (1 : 10000, Sigma-Aldrich, St. Louis, MO), anti-p21 (1 : 200, Santa Cruz Biotechnology, Dallas, TX), and anti-phosphorylated Rb (1 : 1000, Cell signaling) followed by appropriate second antibodies for 1 hour. Immunoreactive proteins were detected using the enhanced chemiluminescence (ECL) system (GE Healthcare Bio-Sciences Corp., Piscataway, NJ).

### 2.5. BrdU Cell Proliferation Assay

HUVECs were allowed to reach 50–70% confluency and then incubated with BrdU (25 *μ*M) (Sigma-Aldrich) at 37°C for 2 hours. Cells were fixed with 4% paraformaldehyde for 15 minutes, permeabilized with PBS containing 0.3% Triton X-100 for 15 minutes at room temperature, and incubated with 2 M HCl for 30 minutes at 37°C. After DNA denaturation, the remaining HCl was neutralized by incubating cells with 0.1 M borate buffer. Cells were then washed three times with PBS containing 0.05% Tween 20 (PBST), blocked with 2% goat serum in PBST for 30 minutes at 37°C, followed by incubation with a mouse anti-BrdU antibody (1 : 1000, Sigma-Aldrich) for 30 minutes at 37°C. After washing three times with PBST, cells were incubated with Alexa Fluor 488 goat anti-mouse (1 : 400, Invitrogen) for 30 minutes at 37°C and counterstained with DAPI. Cells were imaged using a fluorescence microscope at a magnification of 100x. BrdU positive cells were counted and normalized to the total cells in the same field.

### 2.6. Tube Formation Assay

HUVECs were seeded on Matrigel matrix- (BD Biosciences)- coated 96-well plates at a density of 1.5 × 10^4^ cells/well in the EGM Endothelial Cell Medium without serum and incubated in humidified 5% CO_2_ at 37°C for 2 hours. Cells were washed twice with HBSS and labeled with 8 *μ*g/mL Calcein AM (Invitrogen) in 50 *μ*L HBSS. Plates were incubated for 30 minutes at 37°C. After washing twice with HBSS, cells were imaged using a fluorescence microscope at a magnification of 100x. Tube length was measured using ImageJ software.

### 2.7. RT-PCR Assay

RNAqueous-4PCR Kit (Invitrogen) was used to isolate total RNA, and cDNA was produced by reverse transcription with M-MLV reverse transcriptase (Invitrogen). Quantitative PCR was performed with StepOne PCR system (Invitrogen) using Power SYBR Green. The fold difference in various transcripts was calculated by the ΔΔCT method using Hprt as an internal control [27]. Subsequently, a melting curve, constructed in the range of 60 to 95°C, was used to evaluate the specificity of the amplification products. Primer sequences for human transcripts were as follows: Hprt For-5′-CCT TGG TCA GGC AGT ATA ATC CA-3′; Hprt Rev-5′-GGT CCT TTT CAC CAG CAA GCT-3′; Sirt1 For-5′-TGC GGG AAT CCA AAG GAT AA-3′; Sirt1 Rev-5′-CAG GCA AGA TGC TGT TGC A-3′; Sirt2 For-5′-TCC CAG CGC GTT TCT TCT-3′; Sirt2 Rev-5′-ACC AGG AGG AGG TCC ACC TT-3′; Sirt3 For-5′-ACA TCG ATG GGC TTG AGA GAG T-3′; Sirt3 Rev-5′-CAT GAG CTT CAA CCA GCT TTG A-3′; Sirt4 For-5′-CCT CTT GGT GGT GGG ATC AT-3′; Sirt4 Rev-5′-CAG GCA GTG AGG ATA AAC CTG TAA-3′; Sirt5 For-5′-CTC GCC CAC TGT GAT TTA TGT C-3′; Sirt5 Rev-5′-GCT GCT GGG TAC ACC ACA GA-3′; Sirt7 For-5′-CGT CCG GAA CGC CAA ATA-3′; Sirt7 Rev-5′-ACG CTG CCG TGC TGA TTC-3′.

### 2.8. Statistical Analysis

Data are presented as mean ± standard deviation. Group differences were evaluated with one way ANOVA followed by post hoc Student's *t*-test using the Student-Newman-Keuls Method. Results were considered significant if *P* < 0.05. Data shown are representative of at least three independent experiments.

## 3. Results

### 3.1. Oxidative Stress Reduces Sirt6 Protein in Endothelial Cells

**Figure 1:**
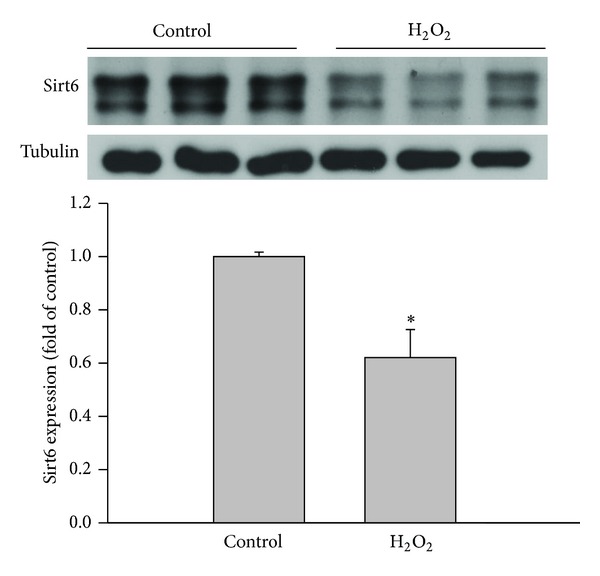
H_2_O_2_ decreases Sirt6 expression in HUVECs. After human umbilical endothelial cells (HUVECs) were incubated in the absence or presence of 75 *μ*M H_2_O_2_ for 4 days, Sirt6 protein was measured by Western blot and normalized to control (*n* = 3). Tubulin was used as loading control. _ _*P < 0.05 compared to control.

H_2_O_2_ is a major reactive oxygen species generated during oxidative stress and has been widely used as an oxidative stress inducer in oxidative stress-related research [11, 28–30]. In order to investigate whether Sirt6 has a potential role in endothelial cell dysfunction induced by oxidative stress, we determined amounts of Sirt6 protein in H_2_O_2_-treated human endothelial cells (ECs). Our results showed that Sirt6 protein was significantly reduced (by 38%) in H_2_O_2_-treated ECs compared to vehicle-treated cells (Figure 1).

### 3.2. Sirt6 Overexpression Attenuates Endothelial Cell Senescence Induced by Oxidative Stress

**Figure 2:**
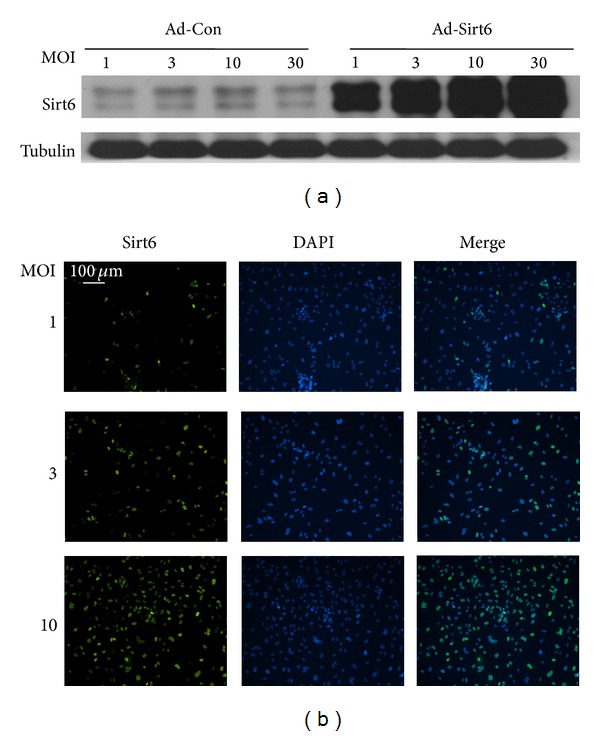
Establishment of overexpression of Sirt6 in endothelial cells. (a) HUVECs were infected with adenovirus carrying Sirt6 gene (Ad-Sirt6) or empty vector (control, Ad-Con) at different multiplicity of infection (MOI). (a) At 24 hrs after infection, cells were lysed and Sirt6 protein level was determined by Western blot. (b) Cells infected with Ad-Sirt6 were stained with DAPI (blue) for nuclei and anti-Sirt6 antibody (green). Images were taken at 100x magnification.

Since Sirt6 expression was downregulated by oxidative stress, we next determined if Sirt6 has a role in oxidative stress-induced endothelial cell senescence. Adenovirus-mediated gene delivery was utilized to introduce Sirt6 into ECs. We observed that when ECs were infected with adenovirus at multiplicity of infection (MOI) from 1 to 30, Sirt6 protein increased in a dose-dependent manner (Figure 2(a)). A MOI of 10 achieved near 100% infection efficiency (Figure 2(b)) and was used in subsequent experiments to overexpress Sirt6 in ECs.

**Figure 3:**
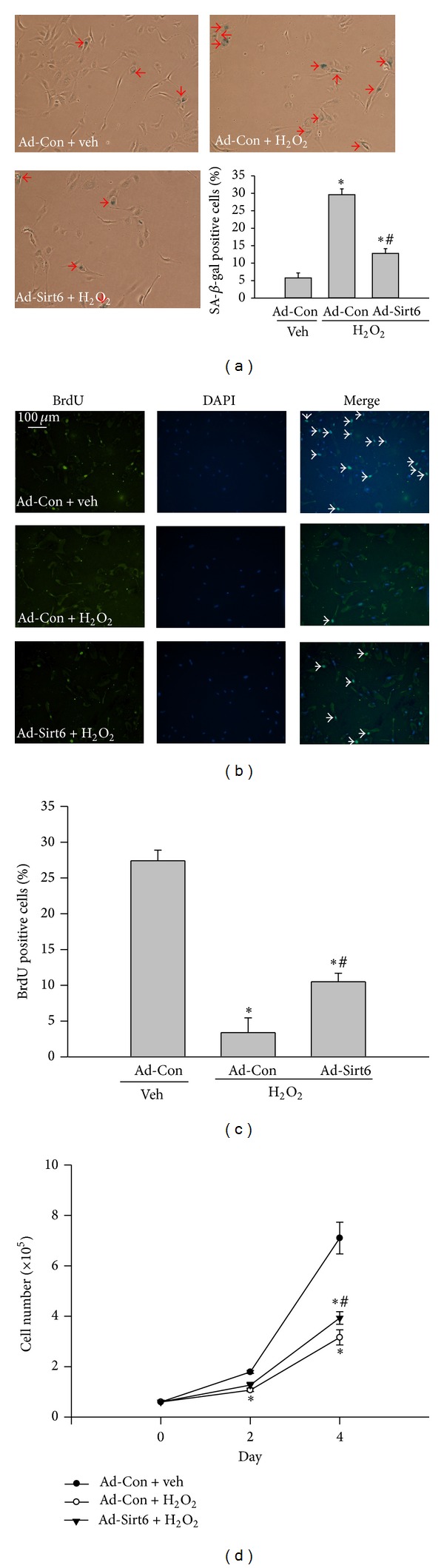
Sirt6 overexpression attenuates endothelial cell senescence induced by H_2_O_2_. ECs were infected with adenovirus carrying Sirt6 gene (Ad-Sirt6) or empty vector (control, Ad-Con) and treated with 75 *μ*M H_2_O_2_ or vehicle (H_2_O) for 4 days. (a) SA-*β*-gal staining was performed using a Senescence Detection kit. Images were taken under a bright-field microscope at a magnification of 100x. Arrows show SA-*β*-gal positive cells (blue cells). The percentage of SA-*β*-gal positive cells versus total cells was determined and quantitative data were shown in the bar graph. (b) Cells were labeled with BrdU followed by staining with DAPI (blue) for nuclei and anti-BrdU antibody (Green). Images were taken at 100x magnification. Arrows show BrdU positive nuclei (green). (c) BrdU positive nuclei (green) and total nuclei (blue) were counted. The percentage of BrdU positive nuclei versus total cell nuclei was calculated and quantitative data were shown in the bar graph. (d) The cell number in each treatment was counted at different times. _ _*P < 0.05 compared with cells infected with Ad-Con and treated with vehicle. _ _^#^ *P* < 0.05 compared with cells infected with Ad-Con and treated with H_2_O_2_.

Senescence-associated *β*-galactosidase (SA-*β*-gal) activity is a characteristic feature of cell senescence. To investigate whether Sirt6 is involved in oxidative stress-induced EC senescence, ECs were infected with adenovirus carrying empty vector (Ad-con) or Sirt6 (Ad-Sirt6) and analyzed for SA-*β*-gal activity after H_2_O_2_ treatment. Compared with vehicle-treated ECs, H_2_O_2_ treatment increased the percentage of SA-*β*-gal positive ECs by 4.1-fold in Ad-con cells. In contrast, in cells infected with Ad-Sirt6, there was only 1.2-fold increase in SA-*β*-gal positive ECs after H_2_O_2_ treatment (Figure 3(a)). As the senescence phenotype suggests a change in growth, we analyzed endothelial cell proliferation by BrdU incorporation. We observed that H_2_O_2_ treatment reduced the percentage of proliferative cells from 27.4% to 3.4%, and overexpression of Sirt6 significantly reversed this effect. The percentage of proliferative cells was increased to 10.5% in cells overexpressing Sirt6 (Figures 3(b) and 3(c)). Similar results were observed by staining cells with the proliferation marker proliferating cell nuclear antigen (PCNA) (data not shown). Consistent with the above markers of EC senescence, EC growth was inhibited by H_2_O_2_ treatment and overexpression of Sirt6 significantly blocked the effect (Figure 3(d)). These results indicate that Sirt6 negatively regulates oxidative stress-induced endothelial cell senescence.

### 3.3. Sirt6 Overexpression Blocks Endothelial Cell Dysfunction Induced by Oxidative Stress

**Figure 4:**
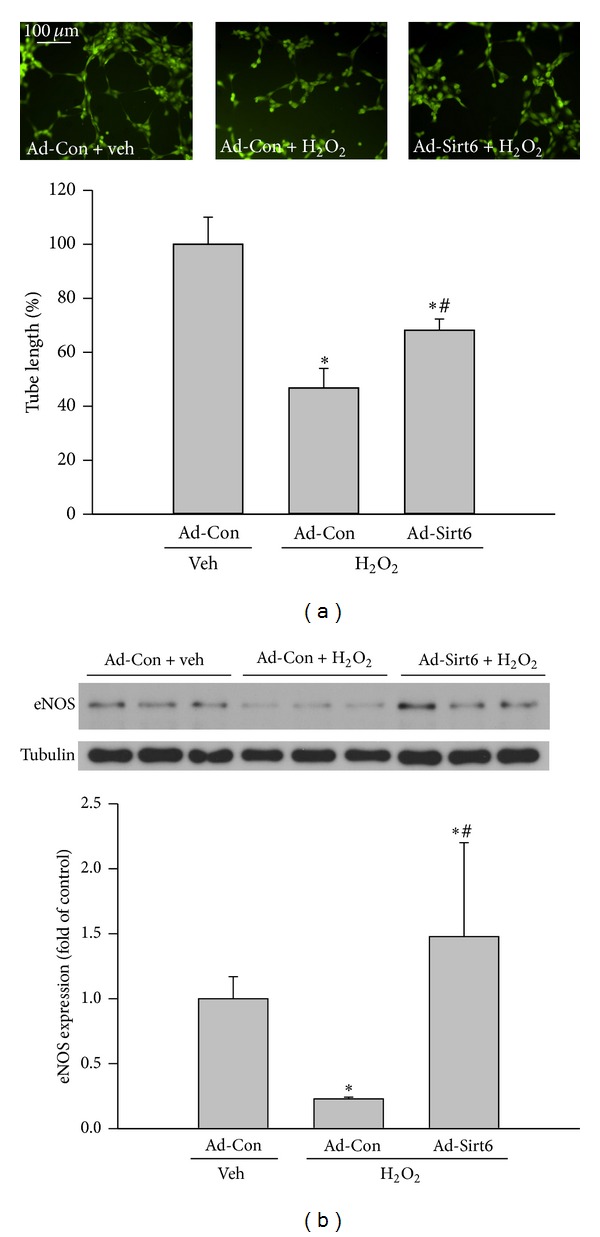
Sirt6 overexpression reduces endothelial cell dysfunction induced by H_2_O_2_. ECs were infected with adenovirus carrying Sirt6 gene (Ad-Sirt6) or empty vector (control, Ad-Con) and treated with 75 *μ*M H_2_O_2_ or vehicle (H_2_O) for 4 days. (a) Cells were subjected to Matrigel-based tube formation assay to determine the angiogenic ability. Images were taken by fluorescence microscopy at 100x magnification and tube length was measured by ImageJ software. (b) eNOS expression in cells was determined by Western blot. _ _*P < 0.05 compared with cells infected with Ad-Con and treated with vehicle. _ _^#^ *P* < 0.05 compared with cells infected with Ad-Con and treated with H_2_O_2_.

EC dysfunction, manifested by reduced production of nitric oxide and impaired angiogenic activity, is a hallmark of vascular diseases. Since EC senescence has been causally linked to EC dysfunction, we analyzed angiogenic activity by tube formation assay and eNOS expression by Western blot in ECs exposed to H_2_O_2_. The total length of tube formation in H_2_O_2_-treated groups was decreased 53% compared with control group. Overexpression of Sirt6 partially restored the angiogenic ability of ECs, with the tube length increased by 46% versus cells treated with H_2_O_2_ (Figure 4(a)). NO is generated by endothelial nitric oxide synthase (eNOS) in ECs. Compared with vehicle-treated control cells, there was a 73% reduction of eNOS protein after H_2_O_2_ treatment (Figure 4(b)). In contrast, H_2_O_2_ treatment did not reduce eNOS protein in Ad-Sirt6 cells. These results suggest that Sirt6 overexpression blocks H_2_O_2_-induced EC dysfunction.

### 3.4. Effects of Sirt6 Are Not Mediated by Other Sirtuin Family Members

**Figure 5:**
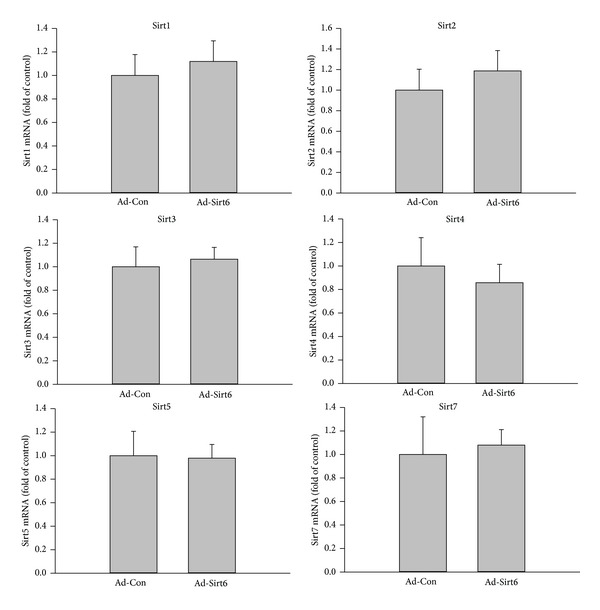
Sirt6 overexpression does not affect the expression of other Sirts. ECs were infected with adenovirus carrying Sirt6 gene (Ad-Sirt6) or empty vector (control, Ad-Con) for 2 days. The levels of other Sirts were determined by quantitative PCR and normalized to control.

Since other members of the sirtuin family have been shown to prevent EC senescence [31], the effects of Sirt6 may be indirect, caused by alterations of other Sirts induced by Sirt6 overexpression. To exclude this possibility, we analyzed mRNA levels of other Sirts in ECs with Sirt6 overexpressed. We observed no significant change in the expression of other Sirts despite elevated Sirt6 by RT-PCR assay (Figure 5). These results indicate that the endothelial cell protective effects of Sirt6 are directly mediated by Sirt6 itself and not indirectly by other members of the sirtuin family.

### 3.5. Moderate Loss of Sirt6 Mimics the Effect of Oxidative Stress

**Figure 6:**
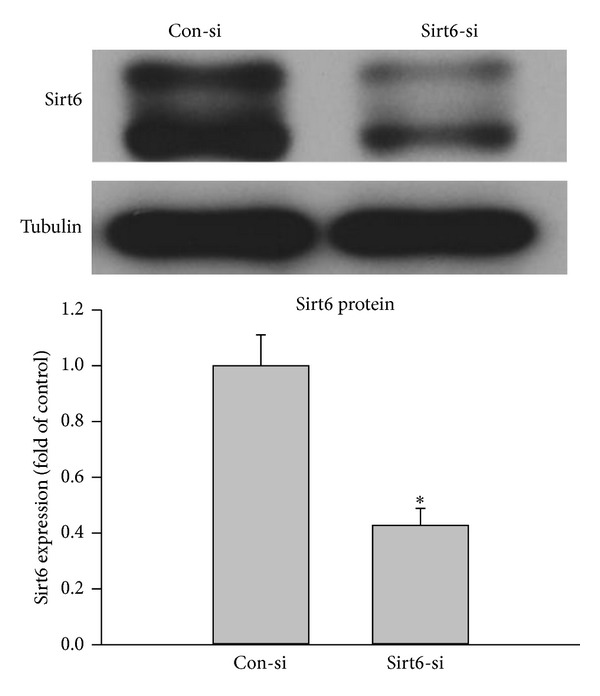
Knockdown of Sirt6 expression in ECs. ECs were transfected with Sirt6 siRNA (Sirt6-si) or Control siRNA (Con-si). 48 hours after transfection, the protein level of Sirt6 was determined by Western blot and normalized to control (*n* = 3). Tubulin was used as loading control. _ _*P < 0.05 compared to control.

**Figure 7:**
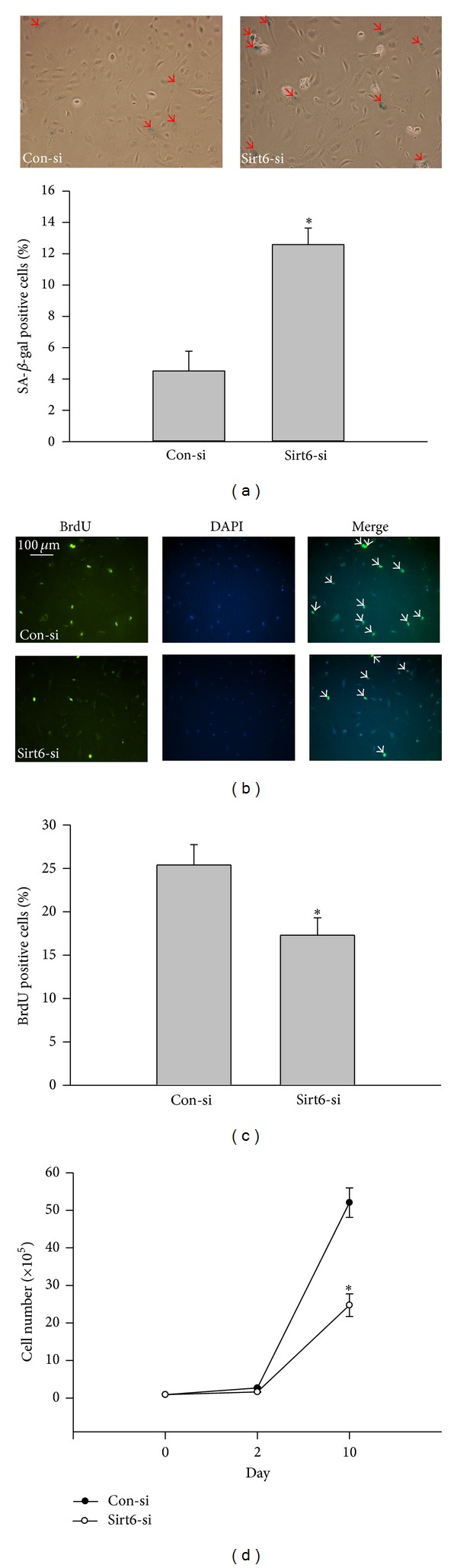
Sirt6 deletion induces endothelial cell senescence. ECs were serially transfected with Sirt6 siRNA (Sirt6-si) or Control siRNA (Con-si) for 10 days. (a) Cells were stained for SA-*β*-gal activity using a Senescence Detection kit. Images were taken under a bright-field microscope at a magnification of 100x. Arrows show SA-*β*-gal positive cells (blue cells). The percentage of SA-*β*-gal positive cells versus total cells was determined and quantitative data were shown in the bar graph. (b) Cells were labeled with BrdU followed by staining with DAPI (blue) for nuclei and anti-BrdU antibody (green). Images were taken under fluorescence microscopy at 100x magnification. Arrows show BrdU positive nuclei (green). (c) BrdU positive nuclei (green) and total nuclei (blue) were counted. The percentage of BrdU positive nuclei versus total cell nuclei was calculated and quantitative data were shown in the bar graph. (d) The cell number after siRNA transfection was counted at different times. _ _*P < 0.05 compared with cells transfected with Con-si.

**Figure 8:**
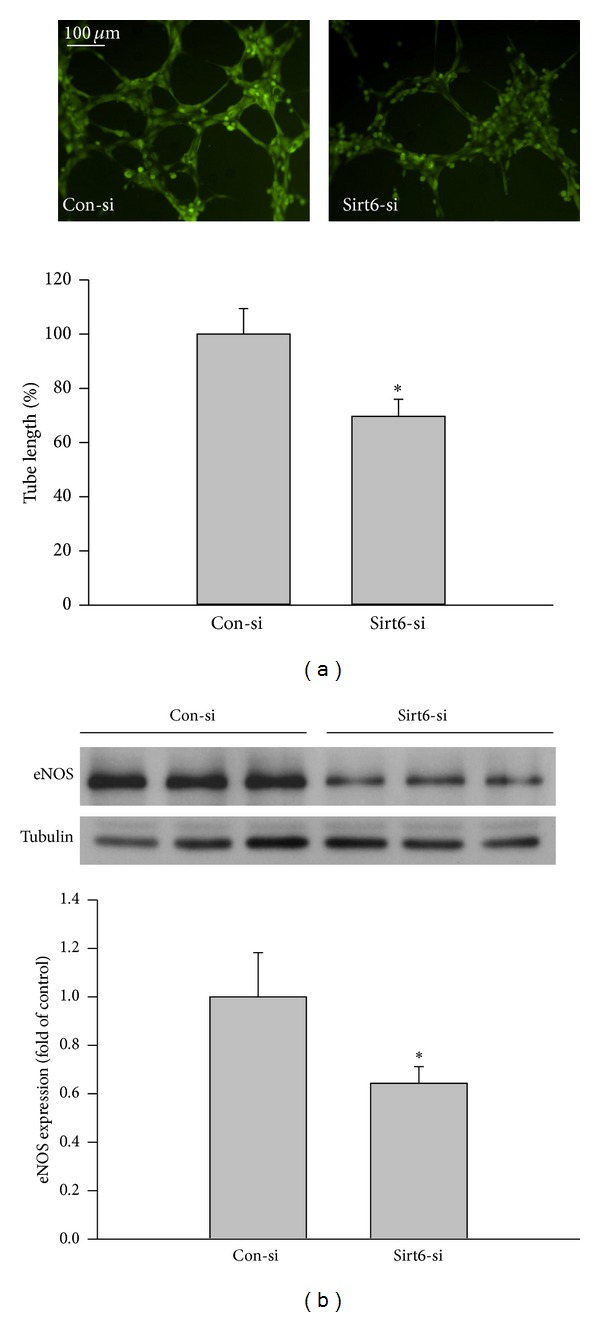
Sirt6 deletion impairs endothelial cell function. ECs were transfected with Sirt6 siRNA (Sirt6-si) or Control siRNA (Con-si). (a) 10 days after transfection, the angiogenic ability of cells was determined by tube formation assay. Images were taken by fluorescence microscopy at 100x magnification and tube length was measured by ImageJ software. (b) eNOS expression in cells was determined by Western blot. _ _*P < 0.05 compared with cells transfected with Con-si.

Since overexpression of Sirt6 can attenuate oxidative stress-induced EC dysfunction and senescence, we tested if moderate knockdown of Sirt6 would mimic effects of oxidative stress. We optimized experimental conditions to reduce endogenous Sirt6 protein level with Sirt6 siRNA (Sirt6-si) by ~60% (Figure 6). At early time points, such as 48 hrs and 72 hrs, we did not observe significant changes in SA-*β*-gal activity and EC growth. However, at 10 days after Sirt6-si RNA transfection, there is a 1.8-fold increase in SA-*β*-gal positive cells, 47% decrease in BrdU positive cells, and 52% decrease in EC growth (Figure 7), suggesting that a moderate knockdown of Sirt6 accelerated EC senescence. Sirt6 siRNA-transfected cells also exhibited signs of EC dysfunction associated with EC senescence, including reduced angiogenic ability and eNOS expression (Figure 8). In summary, these data indicate that a moderate loss of Sirt6 mimics effects of oxidative stress, albeit at a slower pace.

### 3.6. Sirt6 Suppresses Activation of Senescence Pathways Induced by Oxidative Stress

**Figure 9:**
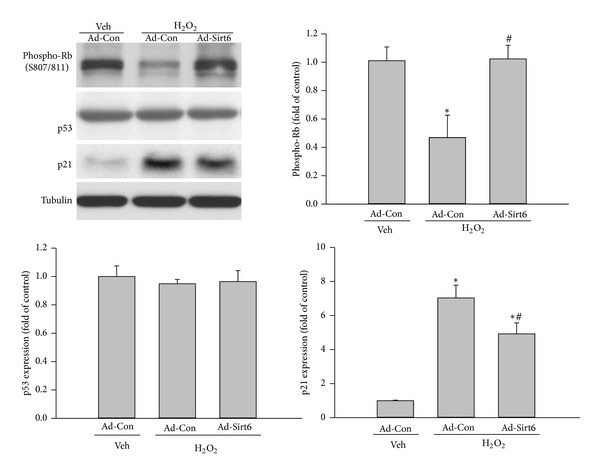
Sirt6 overexpression inhibits the activation of senescence pathways induced by H_2_O_2_. ECs were infected with adenovirus carrying Sirt6 gene (Ad-Sirt6) or empty vector (control, Ad-Con). At 1 day after transduction, cells were exposed to 75 *μ*M H_2_O_2_ or vehicle (H_2_O). At 1 day after H_2_O_2_ treatment, cells were lysed and phosphorylated-Rb, p53, and p21 were determined by Western blot. Tubulin was used as loading control. _ _*P < 0.05 compared with cells infected with Ad-Con and treated with vehicle. _ _^#^ *P* < 0.05 compared with cells infected with Ad-Con and treated with H_2_O_2_.

The retinoblastoma (Rb) protein plays a critical role in the induction of cell senescence. Rb protein is phosphorylated by cyclin-dependent kinases (CDKs), resulting in loss of its ability to bind E2F/DP transcription factor complexes and allowing cells to enter S-phase [32]. However, when the activity of CDKs is blocked by CDK inhibitors, p21Cip1/Waf1/Sdi1, and p16INK4a, Rb protein is hypophosphorylated and activated and inhibits the transcriptional activity of the E2F protein family members [32]. Consequently, cells are arrested in the G1 cell cycle and develop a senescent phenotype [32]. To further elucidate the downstream signaling mechanisms by which Sirt6 inhibits oxidative stress-induced EC senescence, we determined phosphorylation of Rb in cells treated with H_2_O_2_. We found the level of phosphorylated Rb protein was dramatically decreased after H_2_O_2_ treatment (Figure 9), suggesting that activation of Rb protein was involved in H_2_O_2_-induced EC senescence. Consistent with Rb activation, analysis of p21, its upstream activator, also revealed a significant increase in the level of p21 protein. These changes were significantly attenuated by Sirt6 overexpression. p16 protein was undetectable in our experimental conditions (data not shown). These suggest that Sirt6 inhibits oxidative stress-induced EC senescence at least partly through the p21-Rb pathway.

## 4. Discussion

Oxidative stress is a key player in the pathogenesis of diabetic retinopathy (DR) [2, 14–19]. It contributes to endothelial senescence by decreasing NO production, promoting inflammation, and perturbing normal endothelial cell functions. However, molecular mechanisms underlying oxidative stress-induced endothelial senescence remain to be defined. Here, we provide novel evidence that Sirt6, a molecule with antiaging and anti-inflammatory properties, is a target of oxidative stress and is involved in oxidative stress-induced EC senescence. We report that Sirt6 protein was markedly reduced in endothelial cells following treatment with H_2_O_2_ and that overexpressing Sirt6 partially reversed H_2_O_2_-induced EC dysfunction and senescence. This included decreases in EC growth, proliferation and angiogenic ability, loss of eNOS protein, and increases in senescence markers. A partial knockdown of Sirt6 mimicked effects of oxidative stress induced with hydrogen peroxide. Our data, together with a previous observation that lipopolysaccharide-induced reduction of Sirt6 expression is linked to lipopolysaccharide-induced endothelial cell inflammatory reactions [33], highlight the importance of Sirt6 in preventing endothelial dysfunction caused by cellular stress mechanisms.

Similar to a previous publication [34], we observed that knockdown of Sirt6 accelerates endothelial senescence. However, in our study, a significant reduction of EC growth occurred at later time point (10 days) after cells were transfected with Sirt6 siRNA compared to that in Cardus's study (3 days) [34]. This difference between the two studies can be explained by the observation that Sirt6 protein level was reduced by ~60% in our study, mimicking effects of H_2_O_2_, whereas it was decreased by 75% in Cardus et al.'s study [34]. These observations suggest that the phenotype caused by loss of Sirt6 is dose-dependent and a threshold must be reached to induce pathophysiological alternations. Consistent with this suggestion is the observation that Sirt6 heterozygous mice display a normal phenotype regardless of a reduced Sirt6 level [21].

A variety of mechanisms may be involved in the antiendothelial senescence effect of Sirt6. It has been shown that Sirt6 inhibits cell senescence by promoting resistance to oxidative stress-induced DNA damage, suppressing genomic instability, preventing telomere dysfunction, and inhibiting NF-*κ*B mediated expression of age-related genes [21, 23, 35, 36]. In our study, we identified a novel mechanism that involves crosstalk between Sirt6 and Rb protein. Rb, in its hypophosphorylated form, is active and inhibits expression of proteins required for cell-cycle progression by binding to the E2F protein family members and repressing their transcriptional activity [37]. Our results indicate that H_2_O_2_-induced EC senescence was associated with Rb hypophosphorylation and an increase in the expression of p21, an upstream activator of Rb. However, such changes were reversed by Sirt6 overexpression, suggesting Sirt6 may exert antisenescence effect by preventing oxidative stress-induced Rb activation. As p21 is a downstream target of p53 [37], our observations also suggest that Sirt6 is involved in regulation of p53 activity despite the observation that total levels of p53 protein remain unchanged. Previous studies have shown that Sirt1 negatively regulates p53 activity and protects ECs from H_2_O_2_-induced senescence [31]. However, we did not observe changes of other members in the sirtuin family after modulating Sirt6 expression, indicating an independent role of Sirt6 in regulation of EC senescence.

Previous studies have suggested that Sirt6 is involved in protecting cells from oxidative stress by mono-ADP-ribosylating poly(ADP-ribose) polymerase-1 (PARP-1), stimulating PARP1 poly-ADP-ribosylase activity and enhancing repair of double-strand breaks that occur during oxidative stress [38]. Our results suggest that Sirt6 expression could be directly compromised during oxidative stress, and enhanced Sirt6 expression can attenuate oxidative stress-induced endothelial cell senescence. Therefore, agents that can prevent Sirt6 downregulation or enhance Sirt6 activity will be useful in protecting Sirt6 from oxidative stress-induced loss of the normal Sirt6 function during diseases.

Currently, our understanding of mechanisms regulating Sirt6 function is still limited. Sirt6 protein can be ubiquitinated and degraded through the proteasome, and degradation of Sirt6 is substantially prevented when Sirt6 is noncanonically ubiquitinated at K170 by CHIP [39]. Since CHIP protein is downregulated by H_2_O_2_ [40], it is possible that oxidative stress reduces CHIP protein level, resulting in a reduction of Sirt6 protein by accelerating its proteasome-dependent degradation. Alternatively, oxidative stress may regulate Sirt6 expression and function via other posttranslational modifications of the protein, reduction of the level of NAD+, or inhibition of Sirt6 transcription and translation similar to previously described mechanisms regulating Sirt1 function [41]. Future studies are needed to address these possibilities and investigate whether oxidative stress is involved in loss of Sirt6 expression and function in cancer, cardiomyocyte hypotrophy, and inflammation [25, 33, 42].

In summary, our data demonstrate that an oxidative stress-induced decrease in Sirt6 expression has an essential role in oxidative stress-induced endothelial senescence. Given that endothelial senescence is implicated in the development of diabetic vascular complications [8, 9], our results warrant further investigations of the role of Sirt6 in various vascular alternations in DR, such as vascular dysfunction, inflammation, leakage, and pathological angiogenesis.

## References

[1] Zhao Y, Banerjee S, Dey N (2011). Klotho depletion contributes to increased inflammation in kidney of the db/db mouse model of diabetes via RelA (serine)536 phosphorylation. *Diabetes*.

[2] Zhang W, Liu H, Rojas M, Caldwell RW, Caldwell RB (2011). Anti-inflammatory therapy for diabetic retinopathy. *Immunotherapy*.

[3] Elms SC, Toque HA, Rojas M, Xu Z, Caldwell RW, Caldwell RB (2013). The role of arginase I in diabetes-induced retinal vascular dysfunction in mouse and rat models of diabetes. *Diabetologia*.

[4] Mortuza R, Chen S, Feng B, Sen S, Chakrabarti S (2013). High Glucose Induced Alteration of SIRTs in Endothelial Cells Causes Rapid Aging in a p300 and FOXO Regulated Pathway. *PLoS ONE*.

[5] Erusalimsky JD, Skene C (2009). Mechanisms of endothelial senescence. *Experimental Physiology*.

[6] Greider CW (1998). Telomeres and senescence: the history, the experiment, the future. *Current Biology*.

[7] Goligorsky MS, Chen J, Patschan S (2009). Stress-induced premature senescence of endothelial cells: a perilous state between recovery and point of no return. *Current Opinion in Hematology*.

[8] Brodsky SV, Gealekman O, Chen J (2004). Prevention and reversal of premature endothelial cell senescence and vasculopathy in obesity-induced diabetes by ebselen. *Circulation Research*.

[9] Minamino T, Komuro I (2007). Vascular cell senescence: contribution to atherosclerosis. *Circulation Research*.

[10] Foreman KE, Tang J (2003). Molecular mechanisms of replicative senescence in endothelial cells. *Experimental Gerontology*.

[11] Ota H, Eto M, Kano MR (2008). Cilostazol inhibits oxidative stress-induced premature senescence via upregulation of Sirt1 in human endothelial cells. *Arteriosclerosis, Thrombosis, and Vascular Biology*.

[12] Cave AC, Brewer AC, Narayanapanicker A (2006). NADPH oxidases in cardiovascular health and disease. *Antioxidants and Redox Signaling*.

[13] Ushio-Fukai M (2006). Redox signaling in angiogenesis: Role of NADPH oxidase. *Cardiovascular Research*.

[14] Al-Shabrawey M, Bartoli M, El-Remessy AB (2008). Role of NADPH oxidase and Stat3 in statin-mediated protection against diabetic retinopathy. *Investigative Ophthalmology and Visual Science*.

[15] Al-Shabrawey M, Rojas M, Sanders T (2008). Role of NADPH oxidase in retinal vascular inflammation. *Investigative Ophthalmology and Visual Science*.

[16] Rojas M, Zhang W, Xu Z (2013). Requirement of NOX2 expression in both retina and bone marrow for diabetes-induced retinal vascular injury. *PLoS ONE*.

[17] Du Y, Veenstra A, Palczewski K, Kern TS (2013). Photoreceptor cells are major contributors to diabetes-induced oxidative stress and local inflammation in the retina. *Proceedings of the National Academy of Sciences of the United States of America*.

[18] Al-Shabrawey M, Bartoli M, El-Remessy AB (2005). Inhibition of NAD(P)H oxidase activity blocks vascular endothelial growth factor overexpression and neovascularization during ischemic retinopathy. *American Journal of Pathology*.

[19] Madsen-Bouterse SA, Kowluru RA (2008). Oxidative stress and diabetic retinopathy: Pathophysiological mechanisms and treatment perspectives. *Reviews in Endocrine and Metabolic Disorders*.

[20] Ungvari Z, Kaley G, de Cabo R, Sonntag WE, Csiszar A (2010). Mechanisms of vascular aging: new perspectives. *Journals of Gerontology A Biological Sciences and Medical Sciences*.

[21] Mostoslavsky R, Chua KF, Lombard DB (2006). Genomic instability and aging-like phenotype in the absence of mammalian SIRT6. *Cell*.

[22] Lombard DB, Schwer B, Alt FW, Mostoslavsky R (2008). SIRT6 in DNA repair, metabolism and ageing. *Journal of Internal Medicine*.

[23] Kawahara TL, Michishita E, Adler AS (2009). SIRT6 links histone H3 lysine 9 deacetylation to NF-*κ*B-dependent gene expression and organismal life span. *Cell*.

[24] Zhong L, D'Urso A, Toiber D (2010). The histone deacetylase Sirt6 regulates glucose homeostasis via Hif1*α*. *Cell*.

[25] Sundaresan NR, Vasudevan P, Zhong L (2012). The sirtuin SIRT6 blocks IGF-Akt signaling and development of cardiac hypertrophy by targeting c-Jun. *Nature Medicine*.

[26] Xiao C, Wang R, Lahusen TJ (2012). Progression of chronic liver inflammation and fibrosis driven by activation of c-JUN signaling in Sirt6 mutant mice. *The Journal of Biological Chemistry*.

[27] Ameri H, Liu H, Liu R (2014). TWEAK/Fn14 pathway is a novel mediator of retinal neovascularization. *Investigative Ophthalmology & Visual Science*.

[28] Byun M, Jeon K, Choi J, Shim J, Jue D (2002). Dual effect of oxidative stress on NF-*κ*B activation in HeLa cells. *Experimental and Molecular Medicine*.

[29] Fatokun AA, Stone TW, Smith RA (2006). Hydrogen peroxide-induced oxidative stress in MC3T3-E1 cells: the effects of glutamate and protection by purines. *Bone*.

[30] Gutiérrez-Uzquiza Á, Arechederra M, Bragado P, Aguirre-Ghiso JA, Porras A (2012). p38*α* mediates cell survival in response to oxidative stress via induction of antioxidant genes: effect on the p70S6K pathway. *The Journal of Biological Chemistry*.

[31] Ota H, Akishita M, Eto M, Iijima K, Kaneki M, Ouchi Y (2007). Sirt1 modulates premature senescence-like phenotype in human endothelial cells. *Journal of Molecular and Cellular Cardiology*.

[32] Takahashi A, Ohtani N, Hara E (2007). Irreversibility of cellular senescence: dual roles of p16^INK4a^/Rb-pathway in cell cycle control. *Cell Division*.

[33] Lappas M (2012). Anti-inflammatory properties of sirtuin 6 in human umbilical vein endothelial cells. *Mediators of Inflammation*.

[34] Cardus A, Uryga AK, Walters G, Erusalimsky JD (2013). SIRT6 protects human endothelial cells from DNA damage, telomere dysfunction, and senescence. *Cardiovascular Research*.

[35] Michishita E, McCord RA, Berber E (2008). SIRT6 is a histone H3 lysine 9 deacetylase that modulates telomeric chromatin. *Nature*.

[36] Natoli G (2009). When Sirtuins and NF-*κ*B Collide. *Cell*.

[37] Ben-Porath I, Weinberg RA (2005). The signals and pathways activating cellular senescence. *International Journal of Biochemistry and Cell Biology*.

[38] Mao Z, Hine C, Tian X (2011). SIRT6 promotes DNA repair under stress by activating PARP1. *Science*.

[39] Ronnebaum SM, Wu Y, McDonough H, Patterson C (2013). The ubiquitin ligase CHIP prevents SirT6 degradation through noncanonical ubiquitination. *Molecular and Cellular Biology*.

[40] Lee JS, Seo TW, Yi JH, Shin KS, Yoo SJ (2013). CHIP has a protective role against oxidative stress-induced cell death through specific regulation of Endonuclease G. *Cell Death and Disease*.

[41] Salminen A, Kaarniranta K, Kauppinen A (2013). Crosstalk between oxidative stress and SIRT1: impact on the aging process. *International Journal of Molecular Sciences*.

[42] Sebastián C, Zwaans BMM, Silberman DM (2012). The histone deacetylase SIRT6 is a tumor suppressor that controls cancer metabolism. *Cell*.

